# Cement selection criteria for full coverage restorations: A comprehensive review of literature

**DOI:** 10.4317/jced.58671

**Published:** 2021-11-01

**Authors:** Safoura Ghodsi, Sarah Arzani, Mina Shekarian, MohammadMostafa Aghamohseni

**Affiliations:** 1 Dentist, Dental Research Center, Isfahan (Khorasgan) Islamic Azad University of medical sciences, Arghavanieh Blvd, Isfahan, Iran; 2 Dentistry Student, Department of Prosthodontics, Isfahan (Khorasgan) Branch, Islamic Azad University, Isfahan, Iran; 3 Student Research Committee, Islamic Azad University, Isfahan (Khorasgan) Branch, Isfahan, Iran

## Abstract

**Background:**

Proper cement selection in fixed prosthesis plays a determinative role in providing long-term serviceability, retention, caries prevention, and patient satisfaction. This study, reviews different luting agent characteristics and their application based on different clinical situations and different types of full coverage restorations.

**Material and Methods:**

An electronic search was conducted through PubMed, Medline, and Google scholar using following keywords or combinations: restoration, full coverage, PFM, porcelain fused to metal, all ceramic, zirconia, ceramic, casting, fixed partial denture, cement*, dental cement, cement selection, and retention. The most related articles were selected for review.

**Results:**

Choosing a proper luting agent is highly dependent on scientific knowledge regarding the characteristics of restorative materials and luting agents. Conventional cements could be indicated in various situations; however, some restorative materials or clinical situations call for resin-cements to provide predictable retention, support, and durability.

**Conclusions:**

Conscious selection of retentive cement for each type of restoration/material is necessary to provide predictable successful treatment and reduce the potential complications.

** Key words:**Adhesive cement, dental bridgework, dental cements, dental crowns, dental porcelain, prostheses and implants.

## Introduction

Full coverage restorations are among the most prevalent prosthetic treatments used in dentistry. This type of restoration could be indicated in varieties of conditions, ranged from heavily damaged, heavily restored, or cracked tooth to one with aesthetic or positional problems (1-3) and could be fabricated by different materials and methods. Full metal restorations (FM), the strongest and most durable type, has limited applications as more esthetic options have been improved to provide comparable durability, accuracy, and higher acceptance (4,5). Porcelain fused to metal restorations (PFM), the gold standard of prosthetic care, provide acceptable mechanical and esthetic results (6). Rare adverse biological respond, and high long-term survival rate (mean of 75.5% over 20 years) (6-8), have made them the good candidates for restoring highly damaged teeth. However, esthetic appearance, caused by metal framework, overshadowed their applications in high-esthetic area (7). Full-ceramic restorations (FC), introduced less than five decades ago, are esthetic alternatives for PFM (7,9). Good clinical results (survival rate of 74% over 104 months) (10) have candidate them as reliable options for clinical applications. The introduction of computer assisted design-computer assisted manufacturing (CAD-CAM) technology significantly improved the accuracy of prosthetic options and provided the chance of using new types of materials, namely different ceramics with improved characteristics, pre-polymerized resin composites blocks (11), hybrid ceramics, and different alloys.

Single or multiunit tooth- or implant-supported full-coverage prostheses are among the most prevalent prosthetic treatments used in routine dental practices. Retention of indirect restorations, is the main single factor determines their survival and durability (12,13). Although several factors like preparation design, abutment height and width, and surface macro and micro characteristics affect the retention, cementation is prerequisite of retentiveness in indirect restorations. Dental cement is basically used to fill the existing gap between the restoration and prepared tooth and prevent restoration dislodgment by mechanical interlocking (14,15). Since several cementing materials are available today, choosing a proper cement could be confusing even for expert clinicians (12,13,16). Dental cements are categorized in resin-based and acid-based materials (15,17), each has its own characteristics, advantages, and indications (Table 1). Conscious selection of proper cement in each situation could positively affect the quality of long-term dental cares. The present study aimed to make a comprehensive review on available guidance for choosing a proper type of luting cement in each full-coverage restoration type and clinical situation.

**Table 1:**
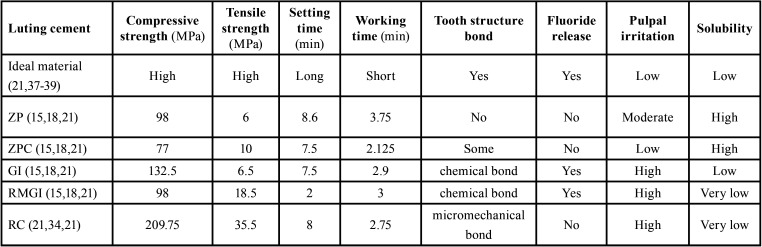
Properties of dental cements. ZP: Zinc Phosphate, ZPC: Zinc Polycarboxylate, GI: Glass ionomer, RMGI: Resin modified glass ionomer, RC: resin cement.

## Material and Methods

An electronic search was performed in PubMed, Medline, and Google scholar using following keyword or combinations: restoration, full coverage, PFM, porcelain fused to metal, all ceramic, zirconia, ceramic, casting, fixed partial denture, cement*, dental cement, selection, comparison, retention. The articles were selected from data bases as well as the related references of selected articles. Using reference management software (Endnote X9; Thomson Reuters), duplicated studies were eliminated. Afterwards, articles were selected based on title/abstract, and full text review. Two independent reviewers evaluated the studies and discussed to reach the same decision. In cases of disagreement, the third reviewer was asked to participate in decision. Peer-reviewed article focused on full-coverage restorations, different cements properties, and cement selection were included, while studies on other types of restorations were excluded, as well as case reports.

## Results

Number of search results for the selected keywords was 8459 (PubMed), 10256 (Google scholar) and 6956 (Scopus). After duplicate removal and title/abstract analysis, 146 studies were selected for full-text review. Finally, 97 studies met the requirement of inclusion/exclusion criteria and were included to be discussed.

Dental cements have a long history. Zinc oxide eugenol (ZOE) was the first luting agent developed in 1850s (18). Zinc phosphate cement was developed thirty years later, and glass ionomer cement was produced in 1972. In 2004 the last generation of luting agent, self-adhesive resin cements, was developed (18). Dental cements could be categorized based on their characteristics. Calcium hydroxide and ZOE cement are used as provisional cements (18). Main disadvantages of ZOE return to obtunding effect on dental pulp, high film thickness, and inhibition effect on resin cements’ polymerization (19,20). Zinc phosphate, zinc polycarboxylate, glass-ionomer, resin-modified glass-ionomer, and resin cements are categorized as long-term definitive cements (20).

Zinc phosphate (ZP) cement is the oldest definitive cement introduced in 1800s, and has a wide range of applications (15,18,21,22). ZP cement has the least biocompatibility (12,21), and no chemical bond to tooth structure (15,18). It has about 98 MPa compressive strength, 6 MPa tensile strength, 13 GPa modulus of elasticity, and a high solubility (0.28%) (15,18).

Zinc Polycarboxylate (ZPC) cement, introduced in 1968 (15), shows molecular adhesion to tooth structure by chemical and van der Waals bond (15,21). ZPC has moderate compressive, and low tensile strength (15,18), and lower solubility and pulpal irritation compared to ZP (15,21). Among the luting agents, ZPC shows the highest initial PH, which results in the highest biocompatibility (18,23).

Glass-ionomer (GI) cement or glass polyalkenoate was first introduced in 1969 (15). The ability of adhesion to tooth and base metal structure, thermal compatibility with enamel, low toxicity, and biocompatibility are among the advantages (24). GI cement has low solubility (21), and toughness (25). However, the most important advantage is fluoride releasing potential, with recharging capability in the oral environment that might play an effective role in caries prevention (15,18).

Resin modified glass ionomer (RMGI) cement, the result of combining resin and GI (18), has improved moisture sensitivity, better mechanical properties, lower solubility, fluoride realizing capability, low translucency (26,27), and the least post cementation sensitivity (28), RMGI cement has a dual mechanism of setting, acid-base reaction, and structural polymerization (18).

Resin cement (RC), the only true adhesive cement (18,29), benefits from cement interlocking in addition to silanization-derived bonding (30). Very low solubility, similar translucency to tooth structure, and various color options have made it the cement of choice in esthetic restorations (21,31). RC could be classified to etch-and-rinse, self-etch, and self-adhesive RC. Etch-and rinse RC was introduced in 1990s, and consists of separate acid etching that is followed by priming/adhesive, and cement application (32,33). This type is technique sensitive, however, provides reliable adhesion, and is the gold standard of adhesive bonding in dental practices (34,31). Self-etch RC combined acid etching and priming in a self-etch primer (32). This type has less technical sensitivity, less dependence on the hydration state of dentin, acceptable dentine bonding (35), but lower enamel bonding strength (25% weaker) (32). Self-adhesive RC, on the other hand, has combined all the components in a single tube to facilitate the cementing procedure (36).

A suitable long-term dental cement should have good biocompatibility, long working and short setting time, low film thickness, low solubility, caries prevention potential, chemical bond to tooth structure, an elastic modulus between tooth structure and restoration, plastic deformation resistance, and accepTable strength and toughness (21,37-39). Table 1 summarizes the properties of dental cements to facilitate the comparison.

## Discussion

-*General guidelines for cement selection*: Selecting proper luting agents should be based on patient requirements, clinical situations, and the restorative material. Having enough information on cements will help the clinician to make a conscious choice; however, there are guidelines that could help to select appropriate cement in each situation: when access and moisture control are difficult to achieve, conventional cements are preferred over resin cements provided that acceptable retention and resistance form is achieved in preparation design (40). Resin cements should be used in partial coverage restorations such as inlays, onlays, and porcelain veneer restorations,41 and are routinely preferred in endodontically treated teeth (42,43). or when reduced retention and resistance are expected in preparation design, height, or taper (40). Preparing the restorations for RC is more demanding since over etching, or over silanization might result in decreased bond strength (44). Different surface preparations with uncertain results or side effects have been suggested to increase resin bond strength for different material (45); however, all these procedures may not be applicable for daily practices in office settings. Resin cements application generally call for more skill, experience, and knowledge.

-*The effect of restorative materials*: a wide range of cement could be used for FM and PFM full coverage restorations. Conventional cements (ZP and GI) could be routinely used in normal single or multi-unit fixed prostheses (46). However, when the situation calls for increased retention (e.g. over-tapered or lower height preparation, long span fixed partial denture, cantilever application, parafunctional or diet habits, and in cases of offset loading on restoration), RMGI or RC could be indicated (47,48). RMGI cement has been suggested as a preferred alternative for other conventional cements considering ease of use, strength, insolubility in mouth environment, and tooth bonding (47). When higher strength and retention are desired, self-curing self-adhesive or pre-encapsulated RC could be the cement of choice (40) with high reported survival rate (49,50).

The important factor for cement selection in ceramic restorations is the composition and structure of ceramic material (41). Ceramic materials are divided into three main categories based on their composition: glass ceramics, polycrystalline (non-glass) ceramics, and hybrid ceramics (51).

Glass ceramic has good esthetic, high biocompatibility, acceptable abrasion and fracture resistance (41), and low mechanical strength for their high glassy content (41,52). Adhesive luting agents have been preferred for cementing these aesthetically appealing ceramics to increase their resistance to fracture (52). Etch-and-rinse type of RC provide higher bond strength, and more durable bonding (37,38); however, other types of RC are also acceptable and well adequate for full coverage restorations (37-39). Conventional cements are contraindicated for this type of ceramics (53). Low glass ceramics, on the other hand, have acceptable esthetic and improved strength (41). Both adhesive or non-adhesive (conventional) cements could be used for cementing full-coverage restorations made from low glass ceramics (54,55). Glass infiltrated ceramics, a branch of low-glass types, have the least glass content and the highest strength and fracture toughness (41). Conventional cements are preferred for this group as the application of acid hydrofluoric (HF) and adhesive bonding does not appear to increase the retention (56); however, RCs could also be used if indicated by clinical situations.

Polycrystalline ceramics are densely sintered oxide ceramics with no glassy content (41,57). Their good resistance to propagation of cracks returns to regularly packed atoms in orderly arrays (58). Polycrystalline ceramics have high strength and toughness, and can be routinely used as frameworks (41) or full-contour restorations. Zirconia (ZrO2), the most well-known branch, was introduced in 1789, and the first paper about its application was published in 1969 (59). Considering high fracture resistance and long-term survival rate, zirconia (Zr) is the most prevalent non-metallic material in fixed prostheses (60). However, Zr shows some problem in adhesion to different substrates, and biomedical applications (60-62). Conventional cements are routinely indicated for full coverage zirconia restorations considering the simple and less demanding procedure. However, sometimes adhesive cements are indicated to achieve better marginal seal, and improved retention and fracture resistance (41,45,60,63-67). In such cases, the application of air abrasion with aluminum oxide or tribochemical silica could effectively increase the bond strength by adhesive resins (68).

Hybrid ceramic were introduced to provide an ideal material with close elastic modulus to the remaining tooth structure while satisfy the esthetic appearance and durability of ceramic materials (69,70). The new hybrid structure resulted in less fragility and superficial hardness that allow easier milling and promising clinical results (71). The evidences do not support using conventional cements in hybrid ceramics (72,73). Resin cements seem to be the main cements of choice for this material (72,73). However, very limited scientific evidences on the clinical success of these materials encourages further studies to be conducted (69).

Resin-composite has also been developed as indirect restorative material in CAD/CAM systems (74). They are less stiff compared to ceramics, that reduces wearing of opposing enamel and facilitates machining process by milling systems (74,75-78); A set of CAD/CAM burs could mill 5-10 ceramic crowns, while for resin-composite blocks this quantity reaches to over 100 crowns (79). Over the time, indirect composite materials have improved in their mechanical properties (66). A previous study showed that alumina airborne abrasion followed by silane and adhesive application could improve retention of resin-composite restorations (80,81).

-*Challenging situations call for increased retention*: Some situations namely extensive destructive caries, abfraction, developmental anomalies, and short height of existing clinical crown are considered challenging for providing adequate retentive and resistance form in abutment teeth (82). The taper (optimal: 6 to 20 degrees) and geometry of prepared tooth as well as tooth surface area and condition, occlusal stresses, and luting agents are considered the main influencing factors in retention (82-84). Certain types of cements provide more retention compared to the others. Should the compromised situations call for increased retention, resin cements could be helpful. It has been reported ZP and GI provides the highest retention among conventional definitive cements, and ZPC shows the least (11).

-*Cement selection in implant restorations*: Fabrication of implant restorations needs a high degree of accuracy since small errors may lead to positional distortion and unfavorable stress on implants (85,86). Although screw-retained restorations show some benefit in retrievability and biocompatibility, cement-retained types are among the prevalent choices for restoring missing teeth that facilitates obtaining passive fit, esthetic, and occlusal accuracy (87). Akca stated that temporary cement provides the least, ZPC provides intermediate, and GI and ZP cause the highest retention among conventional cement applicable in implant restorations (87), and Gultekin clarified that resin cements considerably stablishes the highest retention strength (84). However, considering the implant resistance to caries, temporary cements seem to provide acceptable retention beside the chance of retrieve ability for implant restorations in most clinical situations. Garg and *et al*., stated that Polycarboxylate cement showed the highest retention between Eugenol-free zinc oxide, resin-bonded, ZOE cement, zinc phosphate luting agents (86).

Selecting proper cement is the result of clinician information regarding available luting agents, and the restoration type/material, as well as the patients’ clinical situations. It is mandatory for clinicians to consider the patient and treatment situation and choose the appropriate cement. Table 2 summarizes the results of available studies on different surface treatments and dental cement influence on the restorations’ retention. The present review study attempted to summarize available data on cement selection in one of the most commonly used restorations in dental practices. Introduction of new materials for this type of restoration always call for extensive scientific evaluations to find the best and most effective cement material and ensure long-term clinical results. On the other hand, the evaluation of clinical effectiveness of available new cements specially in compromised or challenging situations requires further research, considering the ever-increasing application of full contour restorations.

**Table 2:**
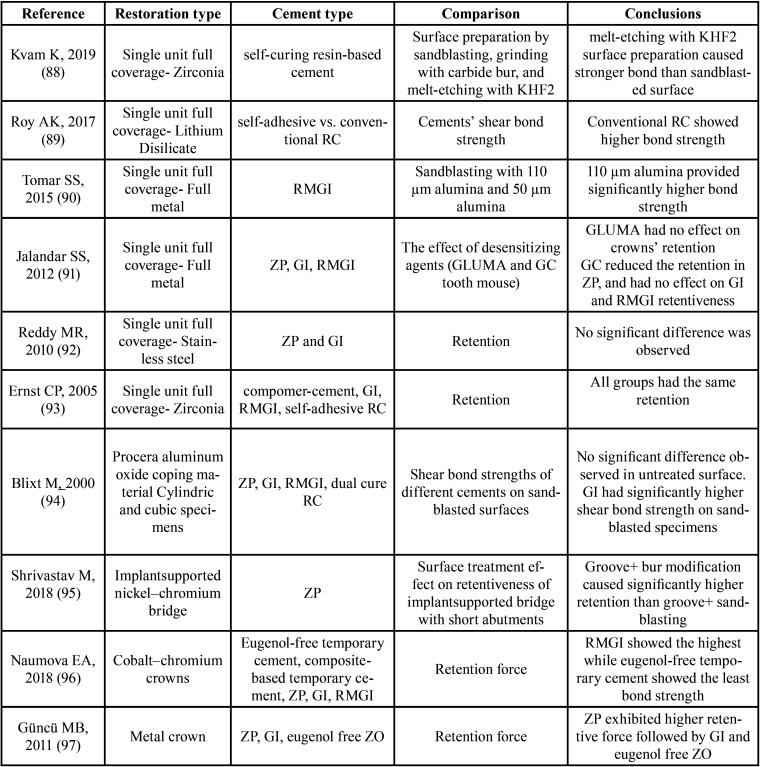
Results of studies on dental cements and surface treatment effects on restorations retention. KHF2: Potassium hydrogen difluoride, RC: Resin cement, RMGI: Resin modified glass ionomer, ZP: Zinc Phosphate, GI: Glass ionomer.

## Conclusions

Reviewing available articles concerning cement selection, the following conclusions can be made:

• Metallic and metal-ceramic full coverage restorations could be cemented by varieties of luting cements and proper cement selection is the result of evaluating tooth preparation, patient clinical situation, and special parafunctional or diet habits.

• Resin cements are indicated for high-glass ceramic restorations to provide reliable adhesive support, while low-glass and polycrystalline ceramics might be cemented by conventional luting agents.

• Resin cements are the only cements indicated in hybrid ceramics.

• Resin cements could be used for full coverage restorations in case of need for increased retention.
